# Contributions to Pharmacy

**Published:** 1854-01

**Authors:** 


*Contributions to Pharmacy.* By John P. Mettauer, M. D., LL. D., of Virginia, Professor of the Principles and Practice of Medicine and Surgery, in the Medical Department of Randolph Macon College.

It will not be denied that the operation of therapeutical agents is essentially influenced by the mode by which they are prepared.

This fact, so generally true, is particularly exemplified in the preparations of cinchona, cantharides, colchicum, guiacum, and several other medicinal substances of which I shall speak presently.

For more than twenty-five years, my attention has been particularly directed to this subject, and, during this period, I have adopted several new methods of preparing some of the articles of the materia medica, and have satisfied myself, by repeated practical trials, that these preparations possessed superior efficacy to those generally employed.

Many years ago I prepared an acetous infusion of catharides,* for blistering purposes. This infusion was first designed for vesicating the scalps of infants, without removing the hair; and its action was very satisfactory. It was applied simply by wetting the surface of the head, and the hair nearest its roots, and then carefully covering the parts with a cabbage leaf, or oiled silk, to prevent the too sudden evaporation of the blistering fluid. When other parts of the body were to be blistered, a thin compress of bibulous paper, or cloth saturated with the infusion, was applied to them, and carefully covered with oiled silk. To insure speedy and effective vesication, I usually re-applied the tincture two or three times, after intervals of half an hour. I found this agent equally as efficient and certain in its action with adults as with infants. It rendered the removal of the hair unnecessary, as it blistered every part of the surface, even when a very thick head of hair existed. This preparation has been used by many of my medical friends, and with entire satisfaction. Within the last ten years, I was induced to prepare an œthereous solution of cantharides† as a vesicant, and have found it far more prompt and certain in its operation than the acetous infusion. It may be applied in the same manner as the latter. Frequently, merely wetting the skin with the solution, without covering the part, will blister; especially in infants. When adults are to be blistered, the preparation should generally be applied with a thin compress, and carefully covered, as already suggested—moistening the compress from time to time, until the skin is decidedly reddened. I have found this by far the most convenient and reliable means of blistering that I have ever employed. This œthereal tincture of cantharides is also an efficient internal remedy. As an emmenagogue and diuretic it has greatly exceeded my expectation. The œthereous menstruum seems not only to promote the operation of the cantharidin upon the genito-urinary organs; but at the same time to guard against strangury. I now use this preparation of cantharides almost exclusively, both externally and internally, when the lytta is indicated, and have done so for seven or eight years.

The remarkable efficacy of the œthereous preparation of the Spanish Fly induced me, five years ago, to employ spirits of nitric œther as a menstruum for cubebs, colchicum, guiacum, squill, ergot, gossypium, sanguinaria, ipecacuanha, digitalis, nux vomica, and some other articles of less importance. The œthereous tincture of cubebs‡ is a most valuable remedy in all the sub-acute inflammations of the bladder, of the urethra, of the uterine cavity, and of the mucous lining of the stomach and intestines. It should be administered in some mucilaginous vehicle.

The tincture of colchicum§ is applicable to the treatment of all of the cases demanding the use of the colchicum, and is decidedly preferable to the vinous seminal tincture now in use, by reason of its tendency to act on the

---

*R. Canth. contus., 2½ oz.; Acid acet., 2 pts. Digest for 14 days, and filter.

†R. Canth. contus., 3 oz.; Spirit. œth. nitric, 2½ pts. Digest for 8 days, and filter.

‡R. Pip. cubeb. contus., 4 oz.; Spirit. œth. nitric, 2 pts. Digest 8 days, and filter.

§R. Sem. colchic. contus., 4 oz.; Spirit. œth. nitric, 2 pts. Digest 10 days, and filter.

urinary system. It is very well adapted to the treatment of sub-acute rheumatism, gout, œdema, and neuraegic rheumatism, especially if the urinary secretion is materially diminished in quantity. In the bloating occasionally connected with the dysmenorrhœa, a combination of this tincture with the œthereous tincture of cantharides, sanguinaria and gum guiacum will be found a most valuable remedy. It should be taken three or four times daily, in an infusion of pine tops, in doses of ten to twenty drops each. The same combination will also be found valuable in the sub-acute stage of gout and rheumatism.

The œthereous tincture of gum guiacum* is superior to the preparations of that article now in general use in the treatment of rheumatism, by reason of its tendency to act on the urinary system; and the same may be said of it as an emmenagogue when there is rheumatic irritation of the uterus as an associate cause of dysmenorrhœa.

The œthereous tincture of squill† is adapted to all cases in which squill is indicated, and is an elegant preparation. In dropsy, œdema of the mucous lining of the larynx, and of the lungs, in asthma, and as an expectorant and diuretic it will be found a most convenient and valuable preparation. A combination of equal parts of this tincture and of the syrup of lobelia inflata taken three or four times daily, in doses of ʒss. to ʒj. each, is the most efficient remedy I have ever used in asthma.

The œthereous tincture of ergot‡ is best suited to cases of inaction or torpor of the uterus connected with debility or exhaustion; it may be used either as an emmenagogue or as a parturient. In uterine hemorrhage, or menorrhagia dependent on debility, or exhaustion of the uterus, it will be found a valuable remedy. Its action upon the uterus is greatly influenced by the œthereous menstruum. It is best to give it in some diuretic vehicle, such as pine tops tea, or flax seed or elm tea; and it may be taken in doses of ʒss. to ʒij., once in four or five hours.

The tincture of gossypium§ is possessed of properties very similar to that of ergot and may be employed in like doses with it, and in similar diseases.

The tincture of sanguinaria¶ is valuable when combined with the tinctures of cantharides, guiacum, colchicum, cubebs, and indeed any other emmenagogue, in the treatment of dysmenorrhœa. It is also a valuable expectorant and diaphoretic in pneumonia, bronchitis, and œdema of the mucous lining of the air passages. It is administered in doses from ʒss. to ʒij., once in three or four hours. This tincture may also be employed alone as a diaphoretic and expectorant.

The œthereous tincture of ipecacuanha‖ is so closely assimilated to the tincture of the sanguinaria in its therapeutical properties, as to be applicable to the treatment of the same diseases. It is an elegant and most convenient preparation. In typhoid fever it will be found far superior to the ipecac pill as a diaphoretic, especially when the tongue is dry and the thirst urgent. It

---

*R. Guiac. gum. resin, 4 oz.; Spirit. œth. nitric, 2 pts. Digest 8 days, and decant.

†R. Seill. maritim. contus., 4 oz.; Spirit. œth. nitric, 2 pts. Digest 8 days, and filter.

‡. Ergot. contus., 2 oz.; Spirit œth. nitric, 1 pt. Digest 10 days, and filter.

§R. Gossypii. herbac, 4z.; Spirit. œth. nitric, 2 pts. Digest for 10 days, and filter.

¶R. Sanguinar. canadens. contus, 4 oz., Spirit. œth. nitric., 2 pts. Digest 8 days and filter.

‖R. Cephæl. ipecac. rad. contus., 2 oz.; Spirit. œth. nitric.; 2 pts. Digest 8 days and filter.

may be used also in typhus fever, or indeed in any febrile affections during the sub-acute stage. This valuable preparation acts both as a diaphoretic and diuretic in these cases, as well as an expectorant.

The œthereous tincture of digitalis* is a far better preparation than the alcoholic, on account of its greater activity; and this it derives chiefly from the œthereous menstruum. In doses from ʒss. to ʒj., in some diuretic infusion, taken three times daily, it will be found well adapted to all such cases as require the foxglove.

The œthereous tincture of nux vomica† is especially indicated in the treatment of seminal debility, or to speak more properly, debility of the generative organs. In this, the gravest of human ills, after such preliminary treatment as may be demanded for the correction of constipation, and prostatic tenderness, this tincture will be found a most excellent means of restoring the erections. It is also valuable in exciting appetite for food, and in the invigoration of the digestive organs. This preparation is well adapted likewise to the treatment of paraplegia, especially when the bladder and rectum are implicated, as well as such other forms of paralysis as demand the nux vomica or its alkaloid. It may be given in doses from ʒss. to ʒiss. three times daily, before or after meals, in some bitter infusion. The cold infusion of wild cherry bark I have generally preferred as the vehicle for it.

The œthereous solutions or tinctures are more readily prepared, requiring to be digested for a less time than the alcoholic, and keep without the least deterioration. They are also adapted to these conditions of the constitution in which alcoholic menstrua would be objectionable.

*Hydrargyrum cum creta.* This valuable preparation of mercury is usually formed by triturating ℥iij. mercury with ℥v. of prepared chalk, until the globules are extinguished. This is a tedious process, and the resulting powder is not of uniform strength, nor is the mercury completely rubbed down. Indeed, it is questionable whether the powder, when apparently well formed, always contains mercury, as a compound may be readily formed by uniting other coloring substances with chalk, to imitate blue mercurial powder; and I think I have met with such imitations several times. The blue powder that I have procured from the shops has generally disappointed me; and for a number of years I have prepared it myself according to the following method:

Take one part of pure starch; eight parts of prepared chalk; and sixteen parts of mercury. Reduce the starch to fine powder. The chalk may now be added, and after being well mixed, the mercury can be united. The powder must next be moistened with water, but not to the extent of wetting it; and the whole rubbed until nearly dry, when the mass should be again moistened and rubbed dry. In this manner the process must be repeated from time to time, as may be convenient, until the powder assumes a uniform bluish appearance. After the chalk seems to be saturated with the mercury, rub the mass perfectly dry, and then moisten it sufficiently to make it adhere to the surface of the mortar by pressing with the pestle. By carefully passing the pestle over the adhering mass, so as to render its surface smooth, the superfluous mercury will now escape from it in small gobules and fall to the bottom of the mortar, and the separation may be facilitated by striking

---

*R. Digital. purp. fal., 1½ oz.; Spirit. œth. nitric., 2 pts. Digest 10 days, and filter.

†R. Nucis vomicæ pulv., 2 oz.; Spirit. œth nitric, 2 pts. Digest 10 days, and filter.

the bottom of the mortar against the table repeatedly, and by pouring the mercury over the surface of the mass where any globules appear. The mercury may now be removed from the mortar; and as soon as the mass becomes sufficiently dry, the trituration must be renewed and continued until the mass becomes a smooth, dry powder. Prepared according to this method, I have used blue powder in my practice more than twenty-five years, and have uniformly found it far more certain in its operation than that obtained from the shops. I prescribe it in the ordinary doses, or nearly so, and yet I am satisfied it is stronger than that in general use. I invariably direct it to be administered nearly dry, united with brown sugar, and to be mixed in a cup, by stirring the powder and sugar together with a straw or the point of a knife. The dose may then be taken into the mouth and swallowed, first with the saliva, and afterwards with a mouthful of water. This powder should never be mixed in a silver spoon, or any other utensil possessing an affinity for mercury, or the powder may be rendered entirely inert; and such an accident once befel a patient of mine, who nearly lost her life before the cause of failure of the medicine in producing its proper effects was discovered.—*Virginia Med. and Surg. Journal.*

